# Global phosphoproteomic profiling of skeletal muscle in ovarian hormone-deficient female mice

**DOI:** 10.1093/geroni/igab046.2532

**Published:** 2021-12-17

**Authors:** Mina Peyton, Tzu-Yi Yang, LeeAnn Higgins, Todd Markowski, Laurie Parker, Dawn Lowe

**Affiliations:** 1 University of Minnesota, Minneapolis, Minnesota, United States; 2 University of Minnesota, University of Minnesota, Minnesota, United States

## Abstract

Dynapenia, the age-related loss of skeletal muscle strength without the loss of muscle mass, significantly impacts the activities and quality of life of the aging population. Studies have shown that dynapenia occurs earlier in females than males in both human and rodent studies. Moreover, in females, estrogen deficiency has been shown to contribute to the loss of skeletal muscle strength as well as blunted recovery of strength after injury. The maintenance of skeletal muscle contractile function is vital to the overall health of women, especially as women live 1/3 of their life in an estrogen deficient state. Reversible protein phosphorylation is an indispensable post-translational modification, playing a key role in signal transduction pathways. Phosphorylation of skeletal muscle proteins have been shown to regulate sarcomeric function, excitation-contraction coupling, energy metabolism, and fiber-type composition. To define the physiological changes in the skeletal muscle phosphoproteome associated with estrogen deficiency, we used an ovariectomy model coupled with mass spectrometry. We identified, in total, 5,424 unique phosphorylation sites and 1,177 phosphoproteins in the tibialis anterior muscle. Ingenuity Pathway Analysis show decreased phosphorylation of contractile proteins and significant predicted inhibition of the upstream kinase, CDK6 (z-score -2.0) in ovariectomized compared to control muscles. Our results suggest that estrogen deficiency remodels the skeletal muscle phosphoproteome which may alter phosphorylation signaling that might contribute to the loss of strength in females.

